# Comparative Evaluation of Spray-Drying Versus Freeze-Drying Techniques on the Encapsulation Efficiency and Biofunctional Performance of Chenpi Extract Microcapsules

**DOI:** 10.3390/foods14101825

**Published:** 2025-05-21

**Authors:** Jiawei Zhao, Xueling Qin, Ying Liu, Qingyun He, Junwei Qin, Fei Shen, Zhenqiang Wu

**Affiliations:** 1 School of Biology and Biological Engineering, South China University of Technology, Guangzhou 510006, China; 202221049328@mail.scut.edu.cn (J.Z.); 202410191285@mail.scut.edu.cn (X.Q.); liuying0199@163.com (Y.L.); 202321049180@mail.scut.edu.cn (Q.H.); qjw0ne@163.com (J.Q.); 2 Pan Asia (Jiangmen) Institute of Biological Engineering and Health, Jiangmen 529080, China; 3 Yiweiyi Biological Manufacturing (Jiangmen) Co., Ltd., Jiangmen 529080, China; 4 Key Laboratory of Functional Foods, Ministry of Agriculture, Guangdong Key Laboratory of Agricultural Products Processing, Sericultural & Agri-Food Research Institute, Guangdong Academy of Agricultural Sciences, Guangzhou 510610, China

**Keywords:** Chenpi extracts microcapsules, spray drying, freeze drying, structural characterization, properties evaluation

## Abstract

Chenpi extracts (CPEs) are highly valued for their rich bioactive compounds and distinctive aromatic properties, but their environmental sensitivity poses stability challenges in food applications. In this study, CPE microcapsules were fabricated using corn peptide as the wall material, and the functional properties of spray-dried microcapsules (SDMCs) and freeze-dried microcapsules (FDMCs) were systematically characterized and compared. The results demonstrate that SDMCs exhibit superior characteristics compared to FDMCs, including reduced moisture content, lower hygroscopicity, enhanced solubility, smaller particle size, and a more uniform microstructure. Both FDMCs and SDMCs showed excellent thermal stability. The SDMCs of CPE encapsulated 93.45% of flavonoids, 90.35% of polyphenols, and 81.32% of sugars from the CPE, while also demonstrating exceptional retention of key terpene volatile compounds, particularly D-limonene (44.63%), γ-terpinene (45.18%), and β-myrcene (40.17%). In contrast, FDMCs exhibited stronger retention of alcohol-based volatile compounds. Furthermore, SDMCs displayed higher antioxidant and hypoglycemic activities, along with improved storage stability. In vitro digestion results reveal that SDMCs provide enhanced protection for CPE flavonoids and polyphenols, achieving bioaccessibility rates of 95.64% and 94.57%, respectively. These findings offer a theoretical basis for optimizing the drying processes in CPE microencapsulation, striking a balance between functional properties and flavor preservation for advanced food applications.

## 1. Introduction

Xinhui Chenpi (CP), a renowned medicinal food homology material, has been extensively utilized in both traditional Chinese medicine and functional food industries for centuries. Chenpi extract (CPE) contains a complex profile of bioactive constituents, notably, flavonoids, polyphenols, and polysaccharides, which contribute to its anti-inflammatory [1], antioxidant [2], anti-tumor [3], and hypoglycemic [4] properties. However, bioavailability and the structure–activity relationships of CPE components require further elucidation to optimize therapeutic potential. Beyond these medicinal properties, CPE is also highly valued for its rich volatile compounds (VOCs) such as terpenes, alcohols, aldehydes, and phenols. This unique combination of bioactive and flavor compounds positions CPE as a multifunctional ingredient for nutraceutical and culinary applications [5]. However, the practical application of CPE is constrained by several critical technological challenges, including low stability, susceptibility to oxidative degradation, poor bioaccessibility, and the loss of aroma components during processing and storage. These physicochemical limitations have significantly impeded the development of CPE-based products in both nutraceutical and food applications.

Encapsulation has emerged as a transformative technology in the food and beverage sector for enhancing the stability and bioavailability of bioactive compounds [6]. This advanced process involves the encapsulation of bioactive compounds within protective wall matrices, thereby minimizing their physicochemical degradation under adverse environmental conditions [7]. Recent studies have demonstrated the effectiveness of microencapsulation in improving the delivery of hydrophobic natural compounds. Sessa et al. [8] developed a peptide-based encapsulation system for curcumin, significantly enhancing its aqueous solubility and oral bioavailability, which may expand its therapeutic potential and clinical utility. In another study, Altunbas et al. [9] reported that microencapsulated resveratrol exhibited superior cellular permeability compared to the free drug, suggesting a promising approach to boost its anticancer efficacy. These findings highlight the role of advanced drug delivery systems in overcoming the inherent limitations of natural compounds, such as poor solubility and low membrane permeability, thereby facilitating their translation into clinical applications.

The selection of appropriate wall materials is crucial, as it directly impacts the encapsulation efficiency and functional performance of the capsules. Ideal food-grade wall materials must satisfy multiple criteria, including processability, low hygroscopicity, and superior stability [10]. Among potential candidates, corn peptide (CT), a bioactive small-molecular-weight polypeptide derived from the enzymatic hydrolysis or microbial fermentation of corn protein, offers distinct advantages. Compared to its native protein counterpart, CT demonstrates higher absorption, solubility, thermal stability, lower viscosity, and antigenicity. Additionally, CT possesses various biological activities, such as antioxidant, antihypertensive, and alcohol metabolism-promoting effects [11]. These exceptional physicochemical and physiological characteristics, combined with its natural origin, have established corn peptide as a highly promising and consumer-accepted ingredient for functional food applications worldwide.

Drying represents a key unit operation in encapsulation processes, serving as the primary method for stabilizing active compounds and extending their shelf life in food systems. However, the selection of drying methodology profoundly impacts final product quality, as evidenced by comparative studies on pomegranate peel extracts processed via hot air drying versus freeze drying [12]. A comprehensive evaluation by Wu et al. [13] of four drying techniques (shade drying, hot air drying, vacuum drying, and freeze drying) revealed that vacuum drying achieved optimal processing efficiency while preserving product morphology and bioactive content. Within industrial applications, freeze drying and spray drying have emerged as the predominant technologies for encapsulating natural compounds, including polysaccharides, proteins, and synthetic polymers [14,15]. Freeze drying preserves thermolabile nutrients by sublimating the solvent under low-temperature vacuum conditions [16], while spray drying utilizes high-temperature atomization for rapid solvent evaporation and forms capsules through instantaneous wall material solidification [17]. Recent studies demonstrate that freeze drying encapsulated CPE effectively preserves bioactive components, improving retention rate, bioaccessibility, and storage stability [14]. However, this technique presents substantial limitations for commercial implementation, including high energy consumption, prolonged processing times, and limited production capacity. Conversely, spray drying offers distinct industrial advantages with lower operating costs, continuous processing capability, and superior scalability [10]. Despite these benefits, current research on spray-dried and freeze-dried microcapsules remains fragmented and lacks a comprehensive analysis of their unique advantages. Therefore, investigating the differences between freeze drying and spray drying in the preparation of CPE microcapsules and emphasizing their respective application advantages is of considerable importance.

In this study, CT was employed as a novel wall material for the microencapsulation of CPE, with particular emphasis on comparing the differential effects of freeze drying and spray drying on the physical properties, bioactive compounds encapsulation, storage stability, antioxidant and hypoglycemic activities, and bioaccessibility of microcapsules. The results will provide novel insights into the technology-specific advantages of different drying methods for CPE microcapsule production, while demonstrating their potential for enhancing both stability and functionality in food matrix applications.

## 2. Materials and Methods

### 2.1. Materials

Aged (5 years) Chenpi was procured from Xinhui District, Jiangmen City, Guangdong Province, China. CT was obtained from Baisheng Food Ingredients Co., Ltd. (Lishui, China). The following analytical-grade reagents were purchased from Aladdin Reagent Co., Ltd. (Shanghai, China): 1,1-diphenyl-2-picrylhydrazyl (DPPH), 2,2′-azino-bis-(3-ethylbenzothiazoline-6-sulfonic acid (ABTS), Trolox, *p*-nitrophenyl-β-D-glucopyranoside (p-NPG), α-amylase, trypsin (≥250 U/mg), and pig bile salt. External standards for gallic acid, rutin, hesperidin, nobiletin, and tangeretin were acquired from Sigma-Aldrich (St. Louis, MO, USA).

### 2.2. Preparation of CPE and Microcapsules

#### 2.2.1. Preparation of CPE

The cleaned, powdered peel was sieved through a 40-mesh screen. Ultrasonic extraction was conducted by suspending the powder in distilled water at a ratio of 1:20 (*m*/*v*) using ultrasonic instrument (KQ-400KDE Kunshan Ultrasonic Instrument Co., Ltd., Kunshan, China) under conditions of 60 °C, 600 W for 0.5 h [18]. The extract was filtered through Whatman no. 4 filter paper, and the extraction process was repeated thrice to pool the filtrates. The combined filtrate, designated as CPE, was stored at 4 °C for subsequent analysis.

#### 2.2.2. Preparation of Microcapsule Solution

The CPE was added to CT powder at a ratio of 1:200 (*m*/*v*), followed by stirring with a magnetic stirrer for 2 h and subsequent overnight hydration. The extract was then subjected to vacuum filtration to obtain a clarified tangerine peel solution, which was stored at 4 °C for subsequent drying processes.

#### 2.2.3. Preparation of Freeze-Dried Microcapsules (FDMCs) of CPE

The stored CPE microcapsule solution was pre-frozen at −80 °C for 24 h and lyophilized using a SCIENTZ-10N freeze dryer (Ningbo Xinzhi Biotechnology Co., Ltd., Ningbo, China) at −58 °C for 48 h to obtain a lyophilized powder [19]. The FDMCs were subsequently homogenized using an agate mortar and pestle to achieve uniform particle size, followed by sieving through a 40-mesh standard screen (425 μm aperture). The processed powder was immediately transferred into a desiccator containing anhydrous calcium sulfate (Drierite™, Hammond, WA, USA) and maintained at 25 ± 2 °C with relative humidity <15% under light-protected vacuum conditions (0.1 MPa) for storage.

#### 2.2.4. Preparation of Spray-Dried Microcapsules (SDMC) of CPE

The stored CPE microcapsule solution was processed using a YC-015 spray dryer (Shanghai Yacheng Co., Ltd., Shanghai, China) under the following operational parameters: feed rate of 8 mL/min, atomization flow rate of 40 mL/min, atomization pressure of 5.0 bar, dry air inlet temperature of 160 °C, and outlet temperature of 120 °C. After completion of drying, the system was cooled until the inlet temperature stabilized below 50 °C [19]. The grinding and storage procedures were performed following the identical protocol described in Section 2.2.3 to maintain methodological consistency throughout this study.

**Figure 1:**
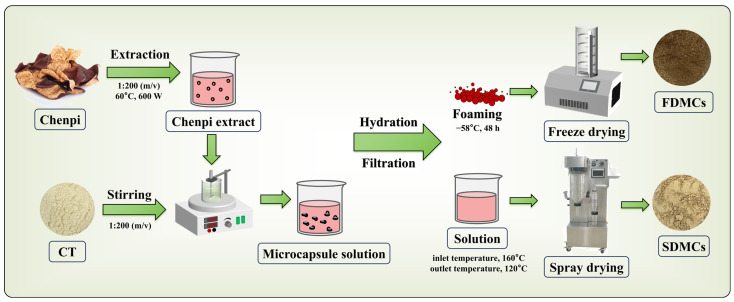
Diagram of processing conditions for microencapsulated CPE powders. CPE: Chenpi extract; FDMCs: freeze-dried microcapsules; SDMCs: spray-dried microcapsules.

The preparation process of CPE microcapsules was shown in Figure 1.

### 2.3. Physical Properties of Microcapsules

#### 2.3.1. Moisture Content

Moisture content was determined by drying 0.5 g of SDMCs or FDMCs in an oven at 105 °C until a constant weight was achieved. The moisture content was subsequently determined using the gravimetric method [20].

#### 2.3.2. Hygroscopicity

Hygroscopicity was evaluated by placing 1 g of SDMCs or FDMCs in a desiccator maintained at 25 °C with a NaCl-saturated solution (75% relative humidity). Following an 8-day equilibration, samples were weighed, and the hygroscopic rate was calculated as grams of moisture adsorbed per 100 g of dry solid (g/100 g) [21].

#### 2.3.3. Bulk Density

The bulk density analysis was conducted as described by Paini et al. [22]. First, 1 g of SDMCs or FDMCs was poured into a 10 mL graduated cylinder, and the volume occupied by the microcapsules was recorded. The bulk density was calculated by measuring the mass of the microcapsules and dividing it by their total volume.

#### 2.3.4. Water Solubility

Solubility was determined following the modified method [23]. Briefly, 0.5 g of microcapsules was dispersed in 50 mL of distilled water and magnetically stirred (420 rpm, 25 °C) for 30 min. The suspension was centrifuged (4000 rpm, 5 min), and the sediment was dried in the oven at 105 °C for 30 min until its weight was constant.

### 2.4. Characterization of Microcapsules

#### 2.4.1. Ultraviolet Spectra

The CT, CPE, FDMC, and SDMC samples were individually dissolved in distilled water and diluted to a uniform concentration of 0.1 mg/mL. The UV–visible (UV–Vis) absorption spectra of the prepared solutions were subsequently recorded using a spectrophotometer (Unico, Franksville, Wisconsin, USA), across the wavelength range of 250–350 nm, with distilled water serving as the blank control.

#### 2.4.2. Particle Size and Polydispersity Index (PDI)

The particle size and polydispersity index of the samples were determined using the laser diffraction method carried out using a Nanosizer ZS90 instrument (ZEN-3690, Malvern Instruments Ltd., Worcestershire, UK). Approximately 50 mg of the sample was dissolved in 50 mL of deionized water under vigorous stirring, followed by 30 s of ultrasonication to ensure complete homogenization. All experiments were performed in triplicate at a constant temperature of 25 °C. The Formula (1) for calculating PDI is as follows

(1) $$\mathrm{PDI}=\frac{d_{90\;}-\;d_{10}}{d_{50}}\;$$

where d_10_, d_50_, and d_90_ represent the equivalent volume diameter when the cumulative volume is 10%, 50%, and 90%, respectively.

#### 2.4.3. Fourier-Transform Infrared (FT-IR) Spectroscopy

The FT-IR spectra were measured using a transmission infrared spectrometer (Sigma, USA) and pressed into sheets (8–10 MPa, 1–2 min). The spectral range is 4000–400 cm^−1^, the resolution is 4 cm^−1^, the measurement temperature is 25 °C, and the cumulative scanning times are 32 times.

#### 2.4.4. X-Ray Diffraction (XRD)

XRD patterns were obtained using an X-ray diffractometer (Empyrean, Malvern Panalytical Ltd., Worcestershire, UK). XRD patterns were collected in the 2θ range of 5–80° at a rate of 4°/min.

#### 2.4.5. Scanning Electron Microscopy (SEM)

The microstructure of the CPE, CT, and microcapsules was observed via SEM (ZEISS, Oberkochen, Germany). Before observation, the sample is affixed to a double-sided tape mounted on the SEM sample tray, placed in a vacuum evaporator, and sprayed with a layer of gold. SEM operates at 5 kV with magnifications of 1K× and 3K×.

### 2.5. Thermal Analysis

#### 2.5.1. Differential Scanning Calorimetry (DSC)

DSC was employed to determine the transition temperature of the dried samples. Approximately 5 mg of each sample was analyzed under a nitrogen purge flow rate of 20 mL/min. The temperature was ramped from 25 °C to 350 °C at a heating rate of 10 °C/min.

#### 2.5.2. Thermal Gravimetric Analyses (TGA), Derivative Thermogravimetric (DTG) Analyses

Thermal properties were performed using a thermal analyzer under a nitrogen inert atmosphere (20 mL/min). The samples were heated from 25 °C to 600 °C at a constant heating rate of 10 °C/min.

### 2.6. Encapsulation Efficiency (EE) of Active Compounds

#### 2.6.1. Quantification of Total Contents of Flavonoids, Polyphenols, and Sugars

Total flavonoid content (TFC) was quantified using the aluminum chloride colorimetric method [24], total polyphenol content (TPC) was determined via the Folin–Ciocalteu assay [25], and total sugar content (TSC) was measured employing the phenol–sulfuric acid method [26]. The results are expressed as rutin equivalents (RE, mg/g) for TFC, gallic acid equivalents (GAE, mg/g) for TPC, and glucose equivalents (GE, mg/g) for TSC.

#### 2.6.2. Quantification of Three Main Flavonoid Components

The contents of hesperidin, nobiletin, and tangeretin were determined via HPLC, which was slightly modified according to Shen et al. [18]. Briefly, 0.1 g of samples was dissolved in 10 mL distilled water and subjected to ultrasonic extraction for 120 min. The resulting suspension was centrifuged at 10,000 rpm for 10 min. Subsequently, 0.5 mL of supernatant was mixed with 0.5 mL anhydrous ethanol and ultrasonicated for 30 min. Prior to analysis, samples were filtered through a 0.22 μm nylon membrane syringe filter.

The EE was calculated indirectly using Formula (2) based on TFC, TPC, TSC, and three major flavonoid components in both the added extract and embedding powder

(2) $$\mathrm{EE}\;\left(\%\right)=\frac{E_{2}\;-\;E_{1}}{E_{0}}\;\times \;100\;$$

where E_1_ represents the content of unencapsulated TFC/TPC/TSC and individual flavonoid components in the microencapsulated powder, E_2_ denotes the total content of TFC/TPC/TSC and individual flavonoid components in the microencapsulated powder, and E_0_ corresponds to the content of TFC/TPC/TSC and individual flavonoid components in the original extract used for encapsulation.

### 2.7. Volatile Organic Compounds Analysis

The differences between CPE, FDCN, and SDCN were determined using head-space solid-phase microextraction combined with gas chromatography mass spectrometry (HS-SPME-GC-MS). The determination method and instrument parameters followed the procedure described by Shen et al. [18]. The identification of volatile organic compounds was conducted using the National Institute of Standards and Technology (NIST) mass spectrometry library and the Wiley mass spectrometry database. Quantification of volatile organic compounds was performed based on the peak area and concentration of cyclohexanone as an internal standard.

### 2.8. Storage Stability

The CPE, FDMC, and SDMC samples were stored under light-protected conditions at 25 °C for 60 days to assess their storage stability. Throughout the study period, TPC, TFC, and TSC were quantitatively analyzed at 15-day intervals according to Section 2.6.1. The retention rate (CR) of each component was determined using the following Formula (3)

(3) $$\mathrm{CR}\;\left(\%\right)=\frac{C}{C_{0}}\;\times \;100\;$$

where C_0_ is TFC/TPC/TSC at the initial stage, and C is TFC/TPC/TSC at various time periods.

### 2.9. Bioactivity Assessment

The bioactivity of FDMCs and SDMCs was evaluated following ultrasonic dissolution in distilled water and dilution to optimal test concentrations. Trolox was used as positive control. The results were expressed in milligrams per gram of DW equivalent to Trolox, or IC_50_, which indicates the amount of sample required to remove 50% of free radicals.

#### 2.9.1. Analysis of Antioxidant Activity

Free radical scavenging ability: the DPPH free radical scavenging rate and ABTS free radical scavenging rate followed the methods by Jo et al. [27], and the free radical scavenging rates were calculated according to the following Formula (4)

(4) $$\mathrm{Radical}\;\mathrm{scavenging}\;\mathrm{rate}\;(\%)=\;[1\;-\;\frac{A_{1}\;-\;A_{2}}{A_{0}}]\;\times \;100\;$$

where A_0_ is the blank absorbance of DPPH/ABTS, A_1_ is the absorbance of the sample, and A_2_ is the absorbance of the sample blank.

FRAP activity analysis: the FRAP activity was determined following the method by Fu et al. [24], and the results are expressed as Trolox equivalent (DW).

#### 2.9.2. Analysis of Hypoglycemic Activity

α-glucosidase inhibition: the inhibition rate of α-glucosidase was determined following the method by Han et al. [28] and was calculated according to the following Formula (5)

(5) $$\mathrm{Inhibition}\;\mathrm{rate}\;\left(\%\right)=[1\;-\;\frac{B_{1\;}-\;B_{0}}{A_{1\;}-\;A_{0}}]\;\times \;100$$

where A_1_ is the absorbance of the mixture of enzyme and pNPG, A_0_ is the blank control group of pNPG, B_1_ is the absorbance of the mixture of the sample, pNPG, and enzyme, and B_1_ is the absorbance of the sample control of PBS that replaces the enzyme.

α-amylase inhibition: the inhibition rate of α-amylase was determined following the method by Raju et al. [29] and was calculated according to the following Formula (6)

(6) $$\mathrm{Inhibition}\;\mathrm{rate}\;\left(\%\right)=[1\;-\;\frac{B_{1}\;-\;B_{0}}{A_{1}\;-{\;A}_{0}}]\;\times \;100$$

where A_1_ is the absorbance of the mixture of enzyme and starch, A_0_ is the starch blank control group, B_1_ is the absorbance of the mixture of sample, starch, and enzyme, and B_1_ is the absorbance of the sample control of PBS that replaces the enzyme.

### 2.10. In Vitro Digestion Simulation

The stability of CPE and dried microcapsules under simulated in vitro digestion conditions was analyzed following the INFOGEST 2.0 method [30] with some modifications. The digestion process was divided into three stages: oral, gastric, and intestinal digestion.

Oral digestion: a mixture of 0.5 g CPE or dried microcapsules and 10 mL of simulated saliva (pH 7.0) containing α-amylase (75 U/mL) was shaken at 180 rpm and 37 °C for 10 min.

Gastric digestion: following oral processing, samples were subjected to simulated gastric digestion by adding 10 mL of simulated gastric fluid (containing 2000 U/mL pepsin, pH 3.0) to the oral bolus. The mixture was incubated at 37 °C and 180 rpm for 2 h, with 1 mL samples collected at 60 and 120 min.

Intestinal digestion: following gastric digestion, the digestate was subjected to intestinal conditions through the addition of 20 mL of simulated intestinal fluid (pH 7.0) containing pancreatic enzymes (100 U/mL), bile salts (10 mM), and CaCl_2_ (0.01 mM). The mixture was incubated at 37 °C and 180 rpm for 2 h, with 1 mL samples collected at 60 and 120 min.

Bioaccessibility assessment: following each digestion phase, the digestate was centrifuged at 10,000 rpm for 5 min and the supernatant was collected and analyzed for TFC and TPC using the methods described in Section 2.6.1. Bioaccessibility was calculated as follows (7):

(7) $$\mathrm{BA}\;\left(\%\right)=\frac{M}{M_{0}}\;\times \;100\;$$

In the formula, M_0_ is TFC/TPC before digestion, and M is TFC/TPC after intestinal digestion.

### 2.11. Data Statistics and Analysis

The experimental data are reported as the mean ± standard deviation values of the three experiments, and SPSS 27 software (SPSS Inc., Chicago, IL, USA) was used for statistical analysis. One-way analysis of variance (ANOVA) and Duncan’s multipole difference test were used to determine the significance of the differences among the samples, and the significance level was *p* ≤ 0.05.

## 3. Results and Discussion

### 3.1. Effect of Drying Process on the Physical Properties and Active Compounds Content of Microcapsules

Moisture content serves as a quality determinant in dried particles, profoundly influencing key physicochemical characteristics including fluidity, bulk density, and color uniformity. As presented in Table 1, SDMCs exhibited significantly lower moisture content (2.74%) compared to FDMCs (3.77%). This 27.3% reduction in moisture content can be mechanistically explained by the synergistic effects of high temperatures in spray drying, which might reduce drying efficiency due to product adhesion in the drying chamber, and which was not observed in freeze drying [31,32]. This suggests that spray drying is more suitable for industrial applications than freeze drying in terms of moisture content control.

Hygroscopicity, a critical functional property defined as the moisture absorption capacity of powdered materials under controlled humidity conditions, fundamentally determines the handling characteristics and storage stability of encapsulated food ingredients [33]. Consistent with previous reports on protein-based encapsulation systems [34], both microcapsule formulations demonstrated favorable hygroscopic properties, with SDMCs exhibiting 15.12% moisture uptake and FDMCs showing 20.11% (Table 1). While FDMCs displayed 33% higher hygroscopicity than SDMCs (*p* ≤ 0.05), both values remain within the acceptable threshold (<25%) for food-grade powders. These results demonstrate that both microcapsules are well-suited for storage and handling, maintaining their structural integrity and quality over time. This highlights their potential for practical applications in the food industry.

The bulk density represents the mass-to-volume ratio of powders in their natural packed state. As quantified in Table 1, SDMCs exhibited 85.71% higher bulk density (0.39 g/cm^3^) compared to FDMCs (0.21 g/cm^3^). This difference can likely be attributed to the smaller particle size and predominantly uniform spherical morphology of the spray-dried microencapsulates, which often contain hollow interiors. These characteristics facilitate closer particle contact and more efficient packing, thereby increasing the bulk density of SDMCs compared to FDMCs [35].

The aqueous solubility, defined as the maximum mass fraction of powder dissolved under standardized conditions, demonstrated exceptional performance for FDMCs and SDMCs, with values of 92.07% and 95.27%, respectively. Both FDMCs and SDMCs demonstrated favorable water solubility, likely attributed to the inherent hydrophilicity of CT powder [36], which facilitated stronger interactions with water molecules and consequently enhanced solubility [37]. Notably, the water solubility of SDMCs was 3.5% higher than that of FDMCs. This enhancement could be attributed to the Maillard reaction between carbohydrates in CPE and amino groups in CT, which was facilitated by the elevated temperature at the spray dryer inlet. The resulting reaction products may have increased the surface polarity of the microcapsules. These findings highlight the effectiveness of both drying processes in producing highly water-soluble CPE microcapsules, with spray drying offering additional advantages in solubility enhancement.

### 3.2. Structural Differences of Microcapsules Under Different Drying Process

#### 3.2.1. Differences in UV–Vis Spectral Peaks

**Figure 2:**
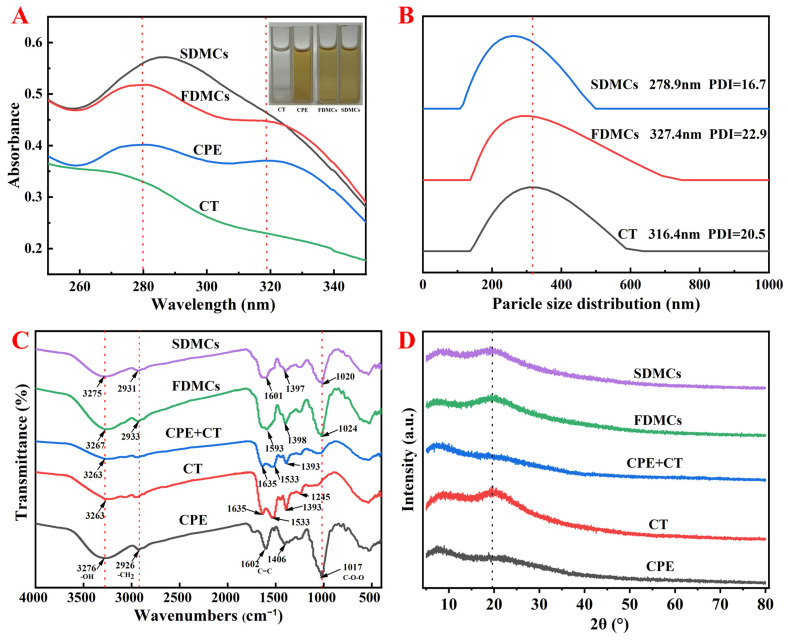
Characterization of CT, CPE, FDMCs, SDMCs, and CPE + CT. CPE + CT: physical mixture of CPE and CT. (**A**): UV–Vis spectrophotometer; (**B**): grain size profile; (**C**): FT-IR spectroscopy. (**D**): XRD. CT: corn peptide; CPE: Chenpi extract; FDMCs: freeze-dried microcapsules; SDMCs: spray-dried microcapsules. CPE + CT: CPE and CT physical mixture.

Changes in UV–Vis spectral peaks can serve as indicators of interactions between materials. As illustrated in Figure 2A, CPE exhibited characteristic absorption peaks at 280 nm and 318 nm. In contrast, FDMCs and SDMCs displayed peaks at 284 nm and 287 nm, respectively, indicating a red shift. The red shift observed for FDMCs was relatively smaller compared to that of CPE, which might be attributed to weaker binding interactions between the CP extract and CT. The spectral profile of FDMCs closely resembled that of CPE, and the disappearance of the 318 nm peak in CPE could be due to the superposition effect of CPE and CT, making it difficult to determine whether new substances were formed during the freeze-drying process. On the other hand, the shift in the peak position of SDMCs from 280 nm to 287 nm, along with a notable change in peak shape, coupled with the expansion of the π-π conjugated system [38] within CPE and CT molecules, indicated that the spray-drying process induces structural modifications. These changes resulted in the formation of new CPE particle structures. These results underscored the distinct impacts of drying processes on the molecular interactions and structural properties of microencapsulated CPE, with spray drying demonstrating a more pronounced effect on inducing structural changes.

#### 3.2.2. Differences in Microcapsule Size Distribution and PDI

Particle size distribution and the polydispersity index (PDI) are critical parameters for evaluating the stability of microcapsules in solution. As shown in Figure 2B, the particle size distribution and PDI of microcapsules in deionized water were characterized. The average particle size of SDMC was 278.9 nm, representing an 11.85% reduction compared to CT, while FDMCs showed an average size of 327.4 nm, corresponding to a 3.48% increase relative to CT. These results indicate that spray-dried particles exhibit smaller dimensions than freeze-dried particles in solution, which is consistent with the SEM observations in this study.

The PDI, a key parameter describing the breadth of molecular weight distribution in polymers, reflects sample heterogeneity—higher PDI values indicate broader size distributions and greater compositional heterogeneity [39]. In the present study, both drying methods yielded PDI values below 0.3, demonstrating their suitability for preparing CP microcapsules. Notably, spray-dried microcapsules showed smaller particle sizes, narrower size distributions, and improved uniformity compared to their freeze-dried counterparts. This enhanced homogeneity contributes to better long-term stability and dispersibility of CPE microcapsules, while also facilitating superior performance upon dissolution of the spray-dried particles.

#### 3.2.3. Differences of Intermolecular Interactions of Microcapsules

FT-IR is a non-destructive technique used to analyze the structure and chemical bond characteristics of substances, making it a powerful tool for studying active molecules and their polymers. FT-IR spectroscopy can also assess the effectiveness of microcapsules. In Figure 2C, the FT-IR spectra of CPE, CT, a physical mixture of CPE and CT powder (CPE + CT), FDMC, and SDMC samples were evaluated and compared in the range of 4000 cm^−1^ to 400 cm^−1^. For CPE, peaks at 1602 cm^−1^ and 1406 cm^−1^ correspond to C=C bond vibrations, primarily from phenols and aromatic compounds [6]. The absorption peak at 1017 cm^−1^ was attributed to the asymmetric stretching vibration of C-O-O bonds on the CP polysaccharide ring, a unique feature of polysaccharides [40]. Peaks at 3276 cm^−1^ resulted from O-H bond stretching, indicating hydroxyl groups in CPE [41]. CT exhibited three typical peaks at 1635 cm^−1^, 1533 cm^−1^, and 1245 cm^−1^, associated with C-O stretching, N-H deformation, C-N stretching, α-helix, β-sheet, and β-turn structures [42]. Comparing FDMCs and SDMCs with CPE + CT, the characteristic CT peaks at 1635 cm^−1^ and 1533 cm^−1^ merged into single peaks at 1593 cm^−1^ and 1601 cm^−1^, respectively, indicating that the interaction between CPE and CT altered the protein’s secondary structure after drying [43]. Upon encapsulation of CPE, the absorption intensity at 3263 cm^−1^ increased due to enhanced hydrogen bonding. While FDMCs and SDMCs exhibited similar characteristic peak profiles, the peak intensity of FDMCs was significantly higher than that of SDMCs. This discrepancy may be attributed to the volatilization of low-boiling-point phenolic compounds (e.g., tangeretin and nobiletin, as identified by HPLC) during the high-temperature spray-drying process [44]. These findings demonstrate the structural changes induced by microcapsules and highlight the distinct effects of freeze drying and spray drying on the molecular interactions and stability of CPE and CT complexes.

#### 3.2.4. Analysis of XRD

XRD is a technique used to analyze the crystal structure of materials based on the interaction between X-rays and the regularly arranged atoms or molecules within a crystal. As shown in Figure 2D, the XRD pattern of the physical mixture of CPE and CT was similar to that of CPE alone, with no discernible peaks corresponding to CT. However, after freeze drying and spray drying, the X-ray diffraction patterns resembled that of CT, with the appearance of CT’s characteristic peaks, indicating that CPE and CT had combined following the drying processes. A comparison of the peak intensities of CT, FDMCs, and SDMCs revealed that the diffraction angles 2θ for FDMCs (19.28°) and SDMCs (19.06°) were slightly shifted compared to that of CT (19.73°). This shift suggested good compatibility between CPE and CT, and indicated that the addition of CPE does not significantly alter the crystalline structure of CT [14]. These findings highlight the effective integration of CPE and CT during the drying processes, preserving the structural integrity of CT while achieving compatibility between the two components.

#### 3.2.5. Morphological Differences of Microencapsulated Microcapsules

**Figure 3:**
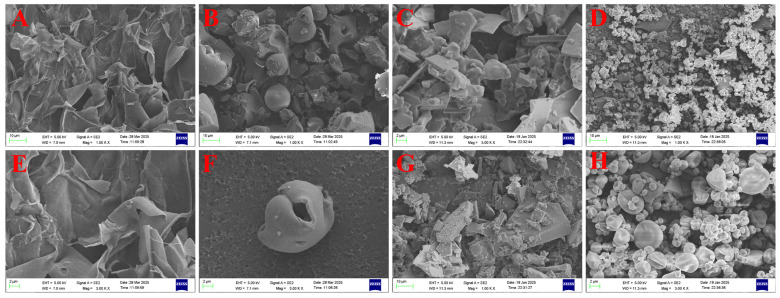
SEM images of CPE, CT powder, FDMCs, and SDMCs. (**A**): CPE (1K×); (**B**): CT powder (1K×); (**C**): FDMCs (1K×); (**D**): SDMCs (1K×); (**E**): CPE (3K×); (**F**): CT powder (3K×); (**G**): FDMCs (3K×); (**H**): SDMCs (3K×). CT: corn peptide; CPE: Chenpi extract; FDMCs: freeze-dried microcapsules; SDMCs: spray-dried microcapsules.

To assess the impact of freeze drying and spray drying on the structural characteristics of Chenpi extract microcapsules, SEM was employed to analyze morphological differences among CPE, CT, FDMC, and SDMC samples. As shown in Figure 3A,E, CPE exhibited fibrous networks with filamentous substances adhering to large polysaccharide matrices. These filamentous structures likely correspond to flavonoids and polyphenols bound to Chenpi polysaccharides. The extensive exposure of polysaccharide surfaces might account for the observed hygroscopicity of CPE, potentially compromising its stability. CT morphology (Figure 3B,F) displayed characteristic concave hollow spheres, a typical feature of protein-based structures [45]. The distinctive surface depressions facilitate effective loading of bioactive compounds from the extract. Freeze-dried microcapsules (FDMCs, Figure 3C,G) presented irregular lamellar structures with membrane-like formations, resulting from foaming effects during lyophilization. This unique architecture enhanced CT’s encapsulation capacity for CPE by trapping active components within the membranous matrix. In contrast, spray-dried particles (SDMCs, Figure 3D,H) demonstrated uniform spherical morphology with smooth surfaces and consistent particle size distribution. The observed concavities and occasional surface pores suggest hollow structures formed by rapid moisture evaporation and internal pressure buildup during high-temperature processing [46]. These structural features promote effective dispersion of core materials [47].

The freeze-drying process generated irregular flakes with increased surface area, facilitating active compound entrapment. Conversely, spray drying produced more homogeneous particles, with the shortened atomization duration contributing to finer particle formation [48]. Notably, the presence of microporous structures in spray-dried particles enhanced core material accessibility while maintaining structural integrity. These findings demonstrated that spray drying yields Chenpi microcapsules with superior morphological characteristics, including enhanced uniformity and structural stability. The morphological advantages correlate with improved functional performance, as will be discussed in subsequent sections analyzing storage stability.

### 3.3. Effect of Drying Processes on the Thermal Analysis of Microcapsules

#### 3.3.1. DSC Analyses

**Figure 4:**
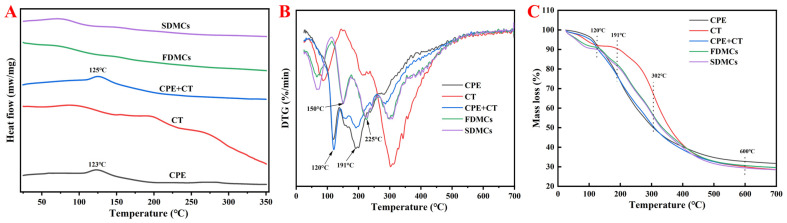
Thermal analysis of CPE, CT, a physical mixture of CPE + CT, FDMCs, and SDMC. (**A**): differential scanning calorimetry; (**B**): differential thermogravimetry; (**C**): thermogravimetric analysis; CT: corn peptide; CPE: Chenpi extract; FDMCs: freeze-dried microcapsules; SDMCs: spray-dried microcapsules. CPE + CT: CPE and CT physical mixture.

Differential scanning calorimetry (DSC) is a widely employed technique in polymer and materials science, providing crucial information regarding the melting/crystallization behavior, glass transition temperature, and thermal stability of materials. The DSC curve of CPE, CT, physically mixed CPE + CT, FDMCs, and SDMCs were analyzed (Figure 4A). The only phase transition observed for CPE was water evaporation at 125 °C. Notably, both FDMCs and SDMCs exhibited no detectable melting/crystallization peaks or glass transitions, making them challenging to characterize using DSC measurements. These findings are consistent with previous reports by Schmidt et al. [49]. Consequently, derivative thermogravimetry (DTG) and thermogravimetric analysis (TGA) were employed to more accurately determine the onset temperature of chemical degradation.

#### 3.3.2. DTG and TGA Analyses

Thermal decomposition behavior of CPE, CT, the CPE + CT physical mixture, FDMCs, and SDMCs was evaluated using derivative thermogravimetry (DTG) and thermogravimetric analysis (TGA), reflecting mass loss under continuous heating. The DTG curves (Figure 4B) revealed that, after microencapsulation with CT, the decomposition peaks of CPE microencapsulation shifted toward higher temperatures, likely due to the protective effect of CT [20]. The initial decomposition temperature of pure CPE was 119.73 °C, while that of the CPE + CT physical mixture was 120.66 °C, indicating that physical blending did not enhance thermal stability. In contrast, after microencapsulation, FDMCs and SDMCs exhibited significantly higher T values of 150.49 °C and 150.58 °C, respectively, demonstrating improved thermal resistance. Additionally, the major decomposition peak of CPE was observed at 191.29 °C, whereas the CPE + CT mixture showed a slight shift to 191.73 °C. Remarkably, after microencapsulation, this peak shifted to 224.78 °C (FDMC) and 222.57 °C (SDMC), confirming that microencapsulation substantially enhances CPE’s thermal stability. This improvement suggests that active compounds in CPE can be effectively incorporated into food matrices without degradation, while also expanding their processing temperature tolerance [50].

The TGA results (Figure 4C) indicate that the thermal decomposition of CPE, CT, and microencapsulated particles occurred in four distinct stages: The first stage is 25–120 °C, which is mainly the volatilization of free water. The second stage is 120–191 °C, which is mainly the loss of bound water and volatile components in CPE. The third stage is 191–303 °C, which is mainly the loss of components in CT and CPE; the chemical bonds in CPE and CT are broken and intermediates such as carbon dioxide are generated. After these three stages, the mass retention of CPE was 50.38%, that of CT was 68.25%, that of the CPE + CT physical mixture was 51.15%, and those for the FDMCs and SDMCs were 57.03% and 56.47%, respectively, indicating that CT has good thermal stability in the range of 25–303 °C, and also significantly improves the heat resistance of FDMCs and SDMCs. Among them, the retention rates of FDMCs and SDMCs are similar. The fourth stage is 303–600 °C, and the intermediate products in this process are further carbonized and decomposed. In general, both drying methods improve the thermal stability of CPE, especially in the range of 25–303 °C. The thermal stability of the microcapsules produced using the two methods is comparable, indicating that both are suitable for the development of tangerine peel-derived products.

### 3.4. Effect of Drying Processes on the EE of Active Components of Microcapsules

**Figure 5:**
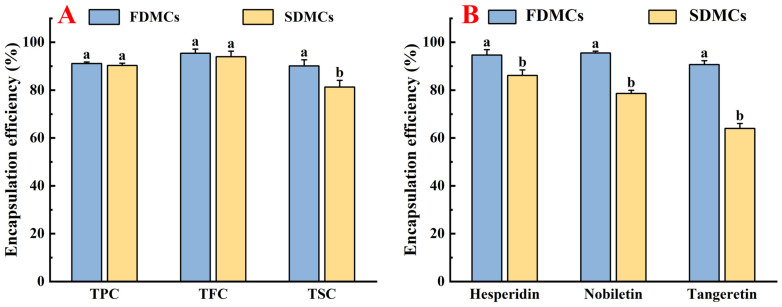
Encapsulation efficiency of FDMCs and SDMCs. (**A**): TPC, TFC, and TSC; (**B**): three main flavonoid components. FDMCs: freeze-dried microcapsules; SDMCs: spray-dried microcapsules. Values represent the mean ± standard deviation (*n* = 3). The letters a and b represent statistically significant differences at (*p* ≤ 0.05).

EE refers to the effectiveness of encapsulating a target substance within a carrier matrix. The preparation of microcapsules containing bioactive compound extracts using two distinct drying processes holds significant importance for the food industry. As illustrated in Figure 5A, the encapsulation efficiencies of TFC, TPC, and TSC in CP microcapsules prepared by freeze drying and spray drying were evaluated. The encapsulation efficiency for FDMCs and SDMCs exceeded 80%, demonstrating effective encapsulation in both cases. Research by Zhang et al. [51] indicated that spray dryers often face limitations in powder recovery due to their small internal dimensions at the laboratory scale. However, industrial-scale dryers, unconstrained by such limitations, are expected to achieve significantly higher yields. Additionally, studies have suggested that foaming during freeze drying creates micropores that enhance the formation of protective films around active compounds [52]. These films effectively retain bioactive substances despite the presence of visible pores, thereby improving encapsulation efficiency. These findings underscore the potential of both drying processes for producing high-quality microcapsules, making them suitable for scalable industrial applications in the food sector.

The encapsulation efficiency of three flavonoids (hesperidin, nobiletin, and tangeretin) in CP, determined with liquid chromatography, were shown in Figure 5B. The encapsulation rate for FDMCs exceeded 90% while, for SDMCs, it ranged from 66.07% to 85.46%. The encapsulation efficiency of hesperidin, nobiletin, and tangeretin in FDMCs was significantly higher than in SDMCs (*p* ≤ 0.05), indicating that high-temperature drying processes are less conducive to retaining these flavonoids. The gradual loss of bioactive compounds during drying was influenced by multiple factors. Compared to larger flavonoid molecules, the relatively low boiling points of small-molecular-weight compounds such as tangeretin (MW: 372.4 g/mol) and nobiletin (MW: 402.4 g/mol) render them more susceptible to volatilization under the elevated temperatures of spray-drying processes. This thermal instability leads to significant losses of these hydrocarbon-based polymethoxyflavones during microencapsulation, ultimately reducing the encapsulation efficiency [19,53]. In freeze drying, water sublimation might create pores in microcapsules, leading to premature release and potential degradation of encapsulated components [54]. However, the foaming effect during freeze drying enhanced film formation around active compounds, effectively retaining them despite visible pores, thereby improving encapsulation efficiency [52]. These findings highlight the trade-offs between drying processes and their impact on bioactive compound retention.

### 3.5. Effect of Drying Processes on the Retention Rate of VOCs in Microcapsules

In this study (Table 2), HS-SPME-GC-MS was employed to analyze the main VOCs in CPE, FDMCs, and SDMCs. A total of 59 volatile components were identified in CPE, with the predominant compounds being D-limonene (4439.36 mg/kg), γ-terpinene (1446.37 mg/kg), β-myrcene (198.86 mg/kg), farnesene (150.45 mg/kg), and 3-carene (114.29 mg/kg). In SDMCs, the retention rates of key terpenes, including D-limonene (44.63%), γ-terpinene (45.18%), β-myrcene (40.17%), farnesene (23.07%), and 3-carene (27.48%), were significantly higher compared to those in FDMCs (*p* ≤ 0.05). Conversely, FDMCs exhibited superior retention of major alcohol volatile components, such as α-terpineol (74.18%), linalool (58.72%), and terpinen-4-ol (36.85%), as well as aldehyde volatile components like capric aldehyde (46.82%), and phenolic volatile components, including nepetalol (56.58%) and thymol (52.43%). These retention rates were significantly higher than those observed in SDMCs (*p* ≤ 0.05).

The higher retention rate of terpenes in SDMCs might be attributed to their encapsulation within CT, which provided protection during the brief high-temperature exposure of spray drying. In contrast, high temperatures could volatilize low-boiling-point substances, such as alcohols and phenols [19], resulting in significantly lower retention rates of these components in SDMCs compared to FDMCs. The mild conditions of freeze drying, however, facilitated the effective retention of low-boiling-point alcohols over extended periods, consistent with the findings by Luo et al. [53]. Overall, spray drying effectively retained the five most abundant alkene flavor components in CP, while freeze drying demonstrated superior retention of alcohols, phenols, and other volatile substances. These findings provide valuable theoretical insights for optimizing the microcapsule drying process to preserve volatile flavor compounds, highlighting the distinct advantages of each drying method for specific applications in the food and flavor industries.

### 3.6. Effect of Drying Processes on the Storage Stability of Microcapsules

**Figure 6:**
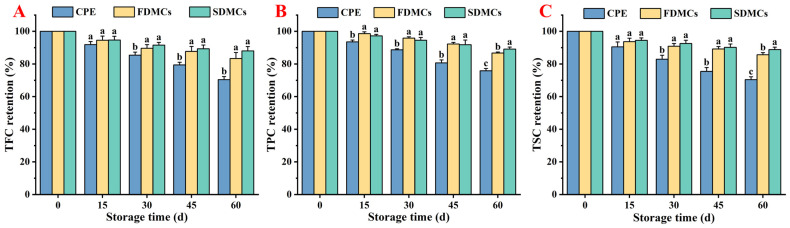
Retention rate of active compounds in FDMCs and SDMCs during storage for 60 d. (**A**): TFC retention; (**B**): TPC retention; (**C**): TSC retention. CPE: Chenpi extract; FDMCs: freeze-dried microcapsules; SDMCs: spray-dried microcapsules. Values represent the mean ± standard deviation (*n* = 3). The letters a, b, and c represent statistically significant differences at (*p* ≤ 0.05).

To evaluate long-term stability, both microencapsulated (FDMC and SDMC) and non-encapsulated CP extracts were stored in sealed aluminum foil bags under controlled conditions (25 °C) for 60 days. The samples were periodically analyzed every 15 days. As illustrated in Figure 6A–C, after 60 days of storage, the TFC of unencapsulated CPE decreased by 33.33%, whereas the encapsulated FDMCs and SDMCs exhibited lower losses of 16.67% and 12.00%, respectively. Similarly, the TPC of unencapsulated CPE declined by 24.20%, compared to reductions of 13.30% and 10.85% for FDMCs and SDMCs, respectively. The TSC of unencapsulated CPE decreased by 29.58%, while the encapsulated FDMCs and SDMCs showed losses of 12.34% and 11.17%, respectively. These findings indicate that microcapsules significantly enhance the storage stability of CP active substances, with spray-dried microencapsulates demonstrating superior retention rates compared to freeze-dried microencapsulates. The CPE formulation exhibited significantly higher TPC loss (24.20%) compared to FDMCs (13.30%) and SDMCs (10.85%) during storage. While CPE retained 75.80% of polyphenols after 60-day storage, this partial preservation may be attributed to physical entrapment of polyphenols within citrus polysaccharide matrices, as confirmed by Figure 3A. However, the substantial exposure of polysaccharide surfaces (Figure 3A) resulted in inadequate protection against oxidative degradation or enzymatic hydrolysis of phenolic glycosides.

Studies have shown that spray drying grape pomace extract maintained TPC levels between 80% and 140% over 45 days, with phenolic compounds potentially degrading or polymerizing to form new compounds [55]. Pelissari et al. [56] found that anaerobic and low-temperature storage conditions favored the stability of spray-cooled lycopene particles. The significant changes in TFC, TPC, and TSC retention rates in unencapsulated CPE might be due to its instability at room temperature, where active components can precipitate or oxidize. In contrast, the addition of peptide powder and drying into microencapsulated particles provided excellent stability for both FDMCs and SDMCs over 60 days in a closed environment.

The higher retention rate of SDMCs compared to FDMCs might be attributed to their lower hygroscopicity, consistent with findings by Dadi et al. [57]. This study demonstrates that microcapsules effectively protect phenolic compounds and flavonoids during storage, with both drying techniques being suitable for preserving CP active substances. These results highlight the potential of microcapsule technology, particularly spray drying, to enhance the shelf life and stability of bioactive compounds in functional foods and pharmaceuticals, offering significant advantages for industrial applications.

### 3.7. Effect of Drying Processes on the Bioactivity of Microcapsules

**Figure 7:**
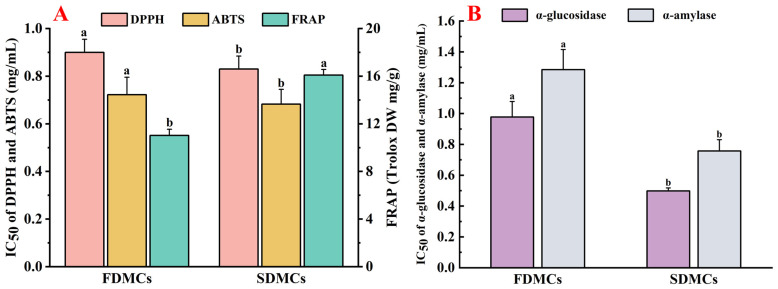
Bioactivities of FDMCs and SDMCs. (**A**): antioxidant activity; (**B**): hypoglycemic activity; FDMCs: freeze-dried microcapsules; SDMCs: spray-dried microcapsules. Values represent the mean ± standard deviation (*n* = 3). The letters a and b represent statistically significant differences at (*p* ≤ 0.05).

In antioxidant activity assays (Figure 7A), the IC_50_ values for DPPH (0.83 mg/mL) and ABTS (0.68 mg/mL) radical scavenging were lower for SDMCs compared to those of FDMCs (0.90 mg/mL) and (0.72 mg/mL), and the FRAP Trolox equivalents were significantly higher for SDMCs (16.09 mg DW/g compared to 11.02 mg DW/g) (*p* ≤ 0.05). In hypoglycemic activity assays (Figure 7B), the IC_50_ values for α-glucosidase (0.50 mg/mL) and α-amylase (0.76 mg/mL) inhibition were also significantly lower for SDMCs compared to those of FDMCs (0.98 mg/mL and 1.29 mg/mL) (*p* ≤ 0.05), indicating that spray-dried microencapsulates exhibit superior antioxidant and hypoglycemic activities compared to freeze-dried microencapsulates. Although the contents of hesperidin, nobiletin, and tangeretin were lower in spray-dried microencapsulates, as shown in Table 1, the flavonoid (2.63 mg/g) and polyphenol (19.60 mg/g) equivalents of SDMCs were significantly higher than those of FDMCs (2.37 mg/g and 17.47 mg/g, respectively).

It has been reported that flavonoids and polysaccharides in CP possess strong antioxidant and hypoglycemic activities, likely due to their ability to regulate the activity of related enzymes [24]. Additionally, the higher water solubility and smaller, more uniform particle size of SDMCs, as observed in SEM results, contribute to enhanced pharmacodynamic properties [58]. These characteristics directly influence the physicochemical properties of the microencapsulates, aligning with the findings of this study. Overall, spray-dried microencapsulates demonstrate superior functional properties, making them more effective for applications requiring high bioactivity and stability. These results highlight the advantages of spray drying over freeze drying in preserving and enhancing the functional properties of bioactive compounds in Chenpi extracts.

### 3.8. Effect of Drying Processes on the Bioaccessibility of Microcapsules

**Figure 8:**
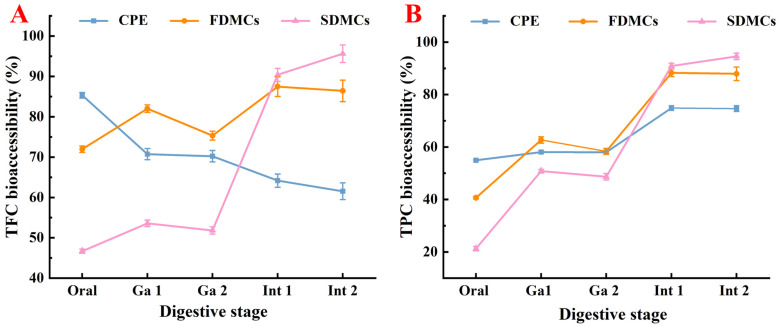
Bioaccessibility of TFC and TPC at different digestion stages of FDMC and SDMC. (**A**): changes in TFC during digestion; (**B**): changes in TPC during digestion. Ga 1: gastric digestion, 1 h; Ga 2: gastric digestion, 2 h; Int 1: intestinal digestion, 1 h; Int 2: intestinal digestion, 2 h. CPE: Chenpi extract; FDMCs: freeze-dried microcapsules; SDMCs: spray-dried microcapsules.

Modeling digestion is essential for understanding how microcapsule methods and materials influence the kinetic release of compounds under gastrointestinal conditions. The results of the in vitro simulated digestion of CPE and the microcapsules are shown in Figure 8, with calculations based on undigested flavonoid content (100%). In the oral digestion stage, unencapsulated CPE exhibited significantly higher TFC retention (85.34%) compared to both microencapsulated forms (*p* ≤ 0.05), suggesting rapid release of free flavonoids from the polysaccharide matrix. During gastric digestion, FDMCs showed superior TFC retention over CPE (*p* ≤ 0.05), demonstrating pH-dependent sustained release from the microcapsules. Unencapsulated flavonoids in CPE underwent acid/enzyme-mediated degradation, while encapsulated forms were protected. SDMCs exhibited significantly lower supernatant content than FDMCs (*p* ≤ 0.05), indicating enhanced gastric stability due to the denser matrix structure. After simulated intestinal digestion, SDMCs achieved 95.64% TFC bioaccessibility through controlled intestinal release, outperforming both CPE and FDMCs (*p* ≤ 0.05). Similar trends occurred for TPC (94.57%), confirming congruent release mechanisms for both compound classes. However, CPE’s polysaccharide-bound flavonoids/phenolics (Figure 3A) showed a delayed release of phenolics (Figure 8B) due to stronger hydrogen bonding with polysaccharides. Faster flavonoid depletion (Table 1), attributable to lower flavonoid-equivalent content in the aqueous phase, reduced steric hindrance for small-molecule diffusion. These results demonstrate that the native polysaccharide matrix in CPE provides only limited protection for Chenpi flavonoids and polyphenols. The data strongly support the necessity of engineered wall materials for creating effective diffusion barriers against acid/enzyme penetration and modulating release profiles through tailored matrix properties.

Smaller particle size promotes more efficient delivery of flavonoids under simulated gastrointestinal conditions, ultimately enhancing the bioaccessibility of CP microencapsulates [59]. Ribeiro et al. [60] demonstrated that spherical microcapsules with smaller particle sizes provide better phenolic protection during in vitro digestion. In conclusion, the TFC and TPC in both microcapsule-drying techniques were significantly higher than in unencapsulated CPE after intestinal digestion, indicating that both techniques enhance the bioaccessibility of CP, with spray drying offering superior microcapsule effects. These findings highlight the potential of microcapsule techniques, particularly spray drying, to improve the stability and bioaccessibility of bioactive compounds under gastrointestinal conditions, making this a promising approach for the delivery of functional compounds in food and pharmaceutical applications.

## 4. Conclusions

This study provides a systematic comparison between freeze-dried microcapsules (FDMCs) and spray-dried microcapsules (SDMCs), revealing significant differences in their physicochemical and functional characteristics. SDMCs demonstrate superior performance characteristics, including enhanced solubility, a homogeneous microstructure, and remarkably high encapsulation efficiency for flavonoids and polyphenols. These advantageous properties contribute to SDMCs’ improved storage stability, antioxidant capacity, and enhanced hypoglycemic activity in vitro. Thermal stability analysis confirmed that both FDMCs and SDMCs exhibit significantly better heat resistance compared to non-encapsulated CPE. In vitro digestion studies demonstrated SDMCs’ superior ability to protect bioactive compounds, increasing the bioaccessibility of flavonoids and polyphenols compared to FDMCs and highlighting the critical importance of microencapsulation for gastrointestinal delivery. Notably, the drying methods showed distinct advantages in flavor retention: SDMCs better preserved volatile terpenes, while FDMCs were more effective in retaining alcohols and phenolic compounds. These findings suggest that spray drying offers distinct advantages for industrial applications requiring product stability and rapid dissolution, whereas freeze drying may be preferable for oxygen-sensitive compounds. The comprehensive data presented here provide valuable guidance for selecting appropriate drying techniques in functional food development and pharmaceutical applications, with specific recommendations based on target compound stability and intended product characteristics.

## Figures and Tables

**Table 1:** Physical properties and active compounds content of microcapsules.

	FDMCs	SDMCs
**Physical properties**		
Moisture content (%)	3.765 ± 0.137 ^b^	2.742 ± 0.256 ^a^
Hygroscopicity (g/100 g)	20.107 ± 1.361 ^b^	15.118 ± 1.317 ^a^
Bulk density (g/cm^3^)	0.211 ± 0.001 ^b^	0.389 ± 0.002 ^a^
Solubility (mg/mL)	92.077 ± 0.460 ^b^	95.270 ± 0.390 ^a^
**Active compounds content**		
TFC (mg DW/g)	2.374 ± 0.054 ^b^	2.632 ± 0.061 ^a^
TPC (mg DW/g)	17.469 ± 0.113 ^b^	19.604 ± 0.201 ^a^
TSC (g DW/g)	0.302 ± 0.009	0.301 ± 0.011

Data are presented as the mean ± SD (*n* = 3). The letters a and b represent statistically significant differences at (*p* ≤ 0.05). FDMCs: freeze-dried microcapsules; SDMCs: spray-dried microcapsules.

**Table 2:** Content and retention rate of VOCs in CPE, FDMCs and SDMCs.

No	Compound Name	CAS	Formula	Structural Type	Molecular Weight	Retention Time	Matching Rate (%)			Content (mg/kg)			Retention Rate (%)	
							CPE	FDMCs	SDMCs	CPE	FDMCs	SDMCs	FDMCs	SDMCs
	**Terpenes**													
1	α-Phellandrene	99-83-2	C_10_H_16_	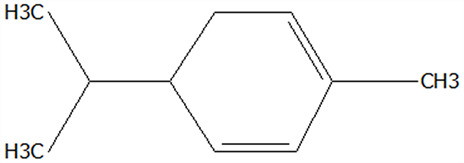	136.23	12.14	84.59	83.73	83.17	25.96 ± 0.31	2.99 ± 0.25	10.68 ± 0.84	11.53 ± 0.96 ^b^	41.13 ± 3.23 ^a^
2	3-Carene	13466-78-9	C_10_H_16_	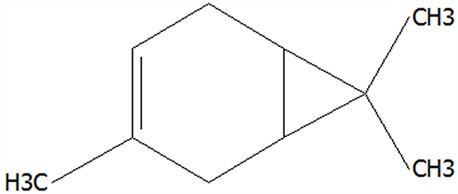	136.23	12.48	88.7	89.07	89.09	114.29 ± 2.14	8.60 ± 0.28	31.41 ± 1.25	7.52 ± 0.24 ^b^	27.48 ± 1.09 ^a^
3	β-Pinene	18172-67-3	C_10_H_16_	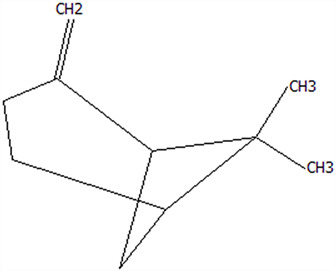	136.23	14.35	83.22	85.05	85.52	97.72 ± 0.94	7.07 ± 0.18	28.23 ± 1.40	7.23 ± 0.18 ^b^	28.89 ± 1.42 ^a^
4	β-Myrcene	123-35-3	C_10_H_16_	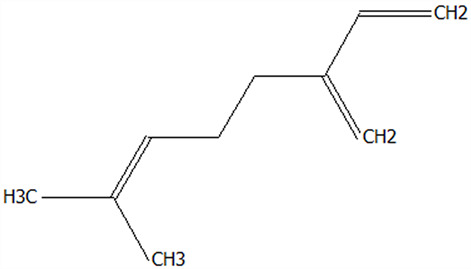	136.23	14.79	82.09	82.1	82.1	198.86 ± 3.08	14.29 ± 0.30	79.88 ± 1.16	7.19 ± 0.15 ^b^	40.17 ± 0.59 ^a^
5	D-Limonene	5989-27-5	C_10_H_16_	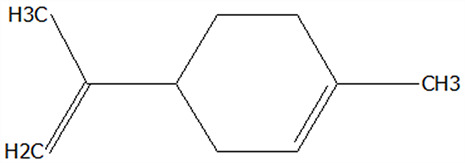	136.23	17.00	81.05	82.32	82.3	4439.36 ± 26.36	533.10 ± 9.16	1981.14 ± 24.47	12.01 ± 0.21 ^b^	44.63 ± 0.55 ^a^
6	γ-Terpinene	99-85-4	C_10_H_16_	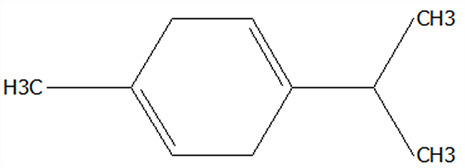	136.23	18.19	75.17	77.43	77.44	1446.37 ± 24.64	132.88 ± 4.06	653.52 ± 13.89	9.19 ± 0.28 ^b^	45.18 ± 0.96 ^a^
7	Terpinolene	586-62-9	C_10_H_16_	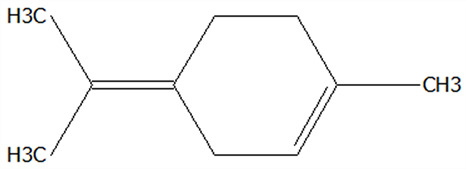	136.23	19.40	81.07	82.96	82.16	69.14 ± 2.13	13.34 ± 0.22	49.89 ± 2.48	19.29 ± 0.32 ^b^	72.15 ± 3.59 ^a^
8	α-Cubebene	17699-14-8	C_15_H_24_	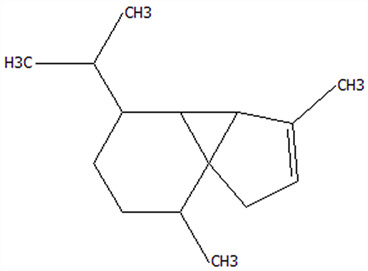	204.35	26.50	81.42		74.31	6.24 ± 0.28	/	3.81 ± 0.16	/	61.12 ± 2.57
9	Copaene	3856-25-5	C_15_H_24_	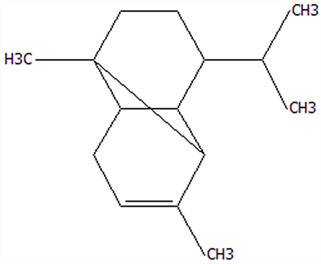	204.35	27.03	74.99	75.57	75.78	30.84 ± 1.42	1.71 ± 0.11	9.56 ± 0.62	5.56 ± 0.36 ^b^	31.00 ± 2.01 ^a^
10	β-Gurjunene	17334-55-3	C_15_H_24_	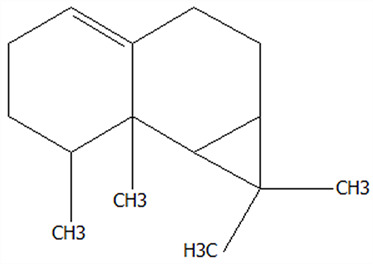	204.35	27.25	82.59	83.03	83.09	19.94 ± 0.10	1.92 ± 0.04	6.91 ± 0.44	9.65 ± 0.20 ^b^	34.68 ± 2.19 ^a^
11	Caryophyllene	87-44-5	C_15_H_24_	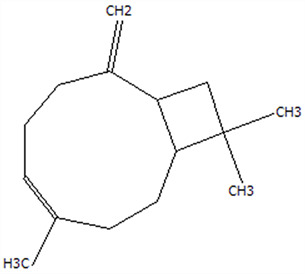	204.35	27.84	85.77	85.95	86	101.65 ± 1.62	3.54 ± 0.17	24.10 ± 1.71	3.48 ± 0.17 ^b^	24.59 ± 1.68 ^a^
12	Farnesene	502-61-4	C_15_H_24_	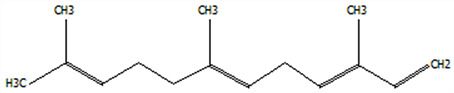	204.35	28.87	81.92	82.08	82.16	150.45 ± 1.94	5.49 ± 0.30	34.70 ± 1.88	3.65 ± 0.20 ^b^	23.07 ± 1.25 ^a^
13	Selinene	473-13-2	C_15_H_24_	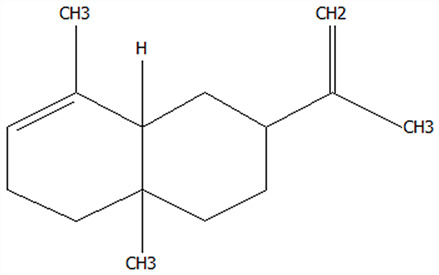	204.35	28.98	79.29		78.5	38.46 ± 1.61	/	7.89 ± 0.35	/	20.52 ± 0.91
14	δ-Cadinene	483-76-1	C_15_H_24_	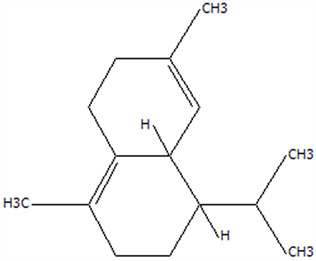	204.35	29.28	78.22		73.05	41.20 ± 2.23	/	10.30 ± 0.93	/	25.00 ± 2.25
	**Alcohols**													
15	Linalool	78-70-6	C_10_H_18_O	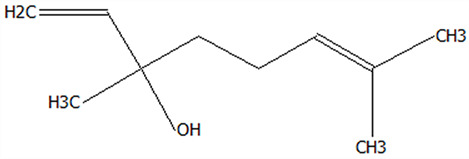	154.25	19.74	79.5	78.59	78.1	23.09 ± 1.84	13.56 ± 0.34	2.04 ± 0.15	58.72 ± 1.48 ^a^	8.84 ± 0.65 ^b^
16	Terpinen-4-ol	562-74-3	C_10_H_18_O	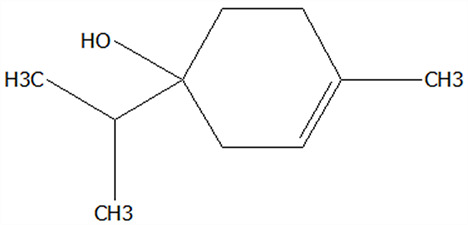	154.25	22.54	76.28	76.05		75.02 ± 2.68	27.65 ± 1.21	/	36.85 ± 1.61	/
17	α-Terpineol	98-55-5	C_10_H_18_O	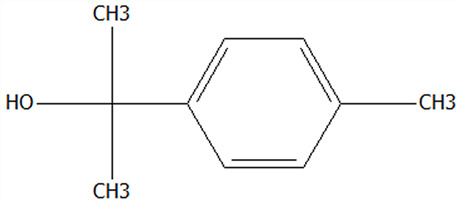	154.25	22.90	76.31	76.51		70.51 ± 1.71	52.31 ± 3.86	/	74.18 ± 5.48	/
	**Aldehydes**													
18	Decanal	112-31-2	C_10_H_20_O	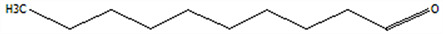	156.27	23.16	78.5	79.95	78.35	38.79 ± 2.96	18.16 ± 1.13	10.90 ± 1.07	46.82 ± 2.92 ^a^	28.11 ± 2.76 ^b^
19	Perillal	2111-75-3	C_10_H_14_O	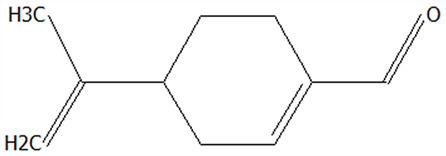	150.22	25.00	83.08	83.49		9.62 ± 0.30	3.60 ± 0.24	/	37.42 ± 2.47	/
20	Undecanal	112-44-7	C_11_H_22_O	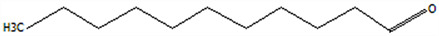	170.29	25.50	75.99			11.30 ± 1.38	/	/	/	/
21	Dodecanal	112-54-9	C_12_H_24_O	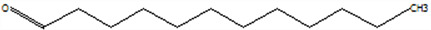	184.32	27.35	79.12	79.93		33.60 ± 2.40	4.14 ± 0.22	/	12.30 ± 0.65	/
	**Phenol**													
22	Thymol	89-83-8	C_10_H_14_O	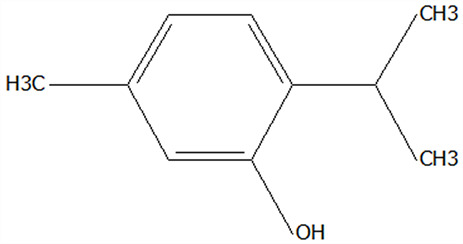	150.22	25.05	71.58	71.82		19.13 ± 0.78	10.03 ± 0.32	/	52.43 ± 1.69	/
23	Carvacrol	499-75-2	C_10_H_14_O	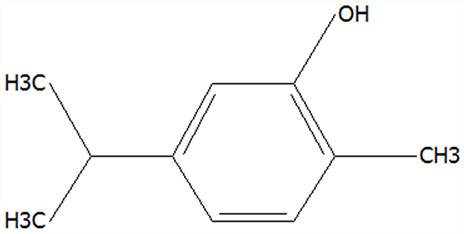	150.22	25.19	74.62	74.95	74.67	75.00 ± 2.74	42.43 ± 1.89	4.01 ± 0.20	56.58 ± 2.53 ^a^	5.34 ± 0.26 ^b^

CAS: Chemical Abstract Service registration number. All names were available in the NIST database. /: absence in sample. The letters a and b represent statistically significant differences at (*p* ≤ 0.05).

## Data Availability

The original contributions presented in the study are included in the article, further inquiries can be directed to the corresponding authors.
